# Verbraucherverschuldung und -überschuldung in Zeiten von COVID-19

**DOI:** 10.1007/s10273-022-3125-4

**Published:** 2022-03-19

**Authors:** Überblick

**Keywords:** G51, E21, D31, I39

## Abstract

Ver- und Überschuldung von Verbraucher:innen engt finanzielles selbstbestimmtes Handeln ein. Aufgabe des Staates ist es, dafür Sorge zu tragen, dass selbst in einer Pandemie ein Mindestmaß an Selbstbestimmung gewährleistet wird. Wo liegt die Grenze für dieses Mindestmaß? Welche Verantwortung sollte eine mit der Wirtschafts- und Sozialpolitik zu verzahnende Verbraucherpolitik übernehmen? 80 Teilnehmer:innen aus Wissenschaft, Verbraucherschutz, Sozialverbänden, Wirtschaft und Ministerien diskutierten am 16.12.2021 auf Einladung des Sachverständigenrats für Verbraucherfragen drei Themen: (1) Konzepte, Entwicklungen, Ursachen der Verbraucherver- und Verbraucherüberschuldung, (2) deren gesellschaftlichen Kontext, sowie (3) Maßnahmen zur Sicherung ökonomischer Selbstbestimmung. Wir veröffentlichen ausgewählte Beiträge.

## Abstract (en)

Consumer debt and over-indebtedness restrict financial self-determination. It is the task of the state to ensure that a minimum level of self-determination is guaranteed even in a pandemic. What is the minimum level? What is the responsibility of a consumer policy that dovetails with economic and social policy? Eighty participants from academia, consumer protection, social associations, business and ministries discussed three topics on 16 December, 2021 at the invitation of the German Council of Consumer Experts: 1) concepts, developments, causes of consumer indebtedness and over-indebtedness; 2) their social context; and 3) measures to secure economic self-determination.

